# General population’s intentions to perform public CPR: a structural equation modeling analysis based on the theory of planned behavior

**DOI:** 10.3389/fpubh.2026.1707826

**Published:** 2026-04-13

**Authors:** Pingjing Zheng, Shenjing Yu, Li Gui, Yixin Wang

**Affiliations:** 1 College of Basic Medicine, Navy Medical University, Shanghai, China; 2 School of Nursing, Naval Medical University, Shanghai, China; 3 Department of Cardiology, State Key Laboratory of Cardiovascular Diseases, Shanghai Institute of Cardiovascular Diseases, Zhongshan Hospital, Fudan University, Shanghai, China

**Keywords:** attitude, behavioral intention, bystander, cardiopulmonary resuscitation (CPR), out-of-hospital cardiac arrest (OHCA), perceived behavioral control, public health, subjective norms

## Abstract

**Background:**

Bystander cardiopulmonary resuscitation (CPR) plays a significant role in boosting survival rates among victims of out-of-hospital cardiac arrests. This study aimed to identify the key factors influencing the public’s intentions to perform CPR based on the Theory of Planned Behavior (TPB).

**Methods:**

A cross-sectional survey was conducted in Shanghai, China, from May 1 to June 20, 2023. Structural equation modeling (SEM) was employed to analyze the relationships between the components of the Theory of Planned Behavior (TPB)—attitude, subjective norms (SN), and perceived behavioral control (PBC)—and CPR intention.

**Results:**

Six hundred eighty-two participants were included in the study. All TPB constructs were found to have significant positive correlations with CPR intention (*r*^2^ > 0.36, *p* < 0.05). The SEM results confirmed that a favorable attitude was a strong predictor of intention (*β* = 0.72). SN were significantly associated with both positive attitudes (*β* = 0.65) and higher levels of PBC (*β* = 0.58). The model exhibited good fit (*χ*^2^/df < 3.0, RMSEA < 0.07, GFI/IFI/AGFI/CFI > 0.90). Furthermore, subgroup analyses revealed significant differences in attitude scores by gender (females: 90.3% ± 15.8% vs. males: 86.6% ± 21.9%, *p* = 0.01) and in intention scores by educational level (senior high school: 88.4% ± 14.3% vs. undergraduate: 78.7% ± 26.0%, *p* = 0.004).

**Conclusion:**

Attitude and SN are the primary determinants of the public’s intention to perform CPR, with SN indirectly shaping intention via attitude and PBC. These findings provide support for TPB-guided interventions aimed at enhancing bystander readiness to perform CPR.

## Introduction

Sudden cardiac arrest (SCA) remains a critical global public health challenge, characterized by its high incidence and dismal survival rates (1). Annually, approximately 5 million deaths worldwide are attributed to SCA, with this burden continuing to rise (2). In China alone, the National Center for Cardiovascular Diseases reports around 550,000 annual SCA cases, over 70% of which occur outside hospital settings (3, 4)—a context where timely intervention is often the sole determinant of survival. Cardiopulmonary resuscitation (CPR) stands as the cornerstone of SCA resuscitation: immediate initiation after collapse can boost survival rates to 67%, while each minute of delay reduces this by 7–10% (5, 6). This underscores the pivotal role of “first responders”—often lay bystanders—in improving resuscitation outcomes. However, China faces a stark gap in public readiness: fewer than 1% of the population has received qualified CPR training, far below the 30–50% prevalence in developed nations (7, 8). Closing this gap requires not only expanding training but also understanding the psychological barriers that prevent trained individuals from acting, making targeted behavioral interventions essential.

While global efforts to promote public CPR engagement—such as scaling education and deploying automated external defibrillators (AEDs)—have shown promise (9–11), significant disparities in bystander response rates persist, particularly in low- and middle-income contexts. Prior research highlights the complexity of factors influencing public CPR performance (12, 13) emphasizing the need to identify core determinants to optimize intervention efficacy. The Theory of Planned Behavior (TPB) offers a robust framework to dissect these determinants: developed by Ajzen, TPB posits that behavioral intention— the proximal predictor of action—is shaped by three constructs: (a) attitude (an individual’s evaluative judgment of the behavior), (b) subjective norms (SN; perceived social pressure to perform the behavior), and (c) perceived behavioral control (PBC; self-efficacy in executing the behavior) (14–17). Widely applied to predict behaviors influenced by social and psychological factors (18), TPB’s utility varies by context, as construct relevance depends on the specific behavior and population studied (19). For high-stakes, time-sensitive actions like bystander CPR—where social expectations, personal confidence, and rapid decision-making intersect—TPB’s constructs may interact in unique ways, necessitating empirical validation.

Against this backdrop, the present study aims to clarify the determinants of public intention to perform CPR and evaluate the predictive validity of TPB constructs in this context. By identifying key psychological drivers, we seek to inform targeted strategies—such as norm-based education or self-efficacy building—to enhance both willingness and capability to act. We test the following hypotheses: (1) Attitude is positively associated with public intention to perform CPR; (2) SN is positively associated with public intention to perform CPR, with indirect effects via (2a) attitude and (2b) PBC; (3) PBC is positively associated with public intention to perform CPR.

## Methods

### Ethical consideration

This study was conducted in accordance with the Declaration of Helsinki and received ethical approval from the Medical Ethics Committee of Naval Medical University (approval number: NMUMREC-2023-022), and strictly followed its guidelines. All participants provided written informed consent prior to enrollment, which detailed the study’s purpose, procedures, minimal risks, and the right to withdraw at any time without consequence. Participation was entirely voluntary. All data were anonymized and stored securely to ensure confidentiality. Participants received no compensation.

### Sample size calculation

Sample size calculation was guided by established recommendations for Structural Equation Modeling (SEM), which suggest a minimum of 10 cases per estimated parameter to ensure stable estimation and adequate statistical power (20, 21). In this study, the 42 items from the validated scale served as an indicator of model complexity, as each item contributes to the estimation of multiple parameters, including factor loadings and error variances. Based on this guideline and accounting for an anticipated 10% attrition rate, the minimum required sample size was calculated as 42 items × 10 cases per parameter × (1 + 0.10) = 462 participants. The final sample of 682 participants exceeded this minimum requirement. This larger sample size enhances the stability of parameter estimates, improves the precision of model fit indices, and provides sufficient statistical power for the planned subgroup analyses, consistent with best practices in SEM. While larger samples can increase the sensitivity of the chi-square test to trivial discrepancies, the obtained sample size remains appropriate for the model’s complexity and strengthens the robustness of our conclusions.

### Participants

A cross-sectional survey was conducted in Shanghai, China, from May 1 to June 20, 2023. Given the goal of efficiently recruiting a diverse sample within the study period, convenience sampling was implemented specifically at popular tourist attractions across different districts of Shanghai, with data collection carried out during peak hours of these sites to capture a broader range of participants. The decision to recruit from popular public sites in Shanghai was guided by two key considerations. First, bystander CPR performance is most critical in high-foot-traffic areas—such as commercial districts, transportation hubs, and tourist zones—where SCA events are more likely to occur in public spaces (as supported by local epidemiological data indicating 38% of community SCAs in Shanghai occur in such locations). Second, these sites attract a demographically diverse population (including residents, commuters, and visitors) with varying levels of exposure to CPR education, which enhances the generalizability of findings to the broader public targeted by community-based CPR interventions.

The inclusion criteria for participants were: (a) aged 18 years or older; (b) capable of independently reading and completing the questionnaire; and (c) having provided written informed consent.

Exclusion criteria were: (a) presence of cognitive or reading disabilities that would hinder questionnaire completion; (b) evident errors in questionnaire responses (e.g., inconsistent answers to logically related items); and (c) a completion time of less than 1 min, as this was deemed indicative of invalid responses.

### Survey tool

The survey was administered in Mandarin Chinese and designed to be accessible to individuals with at least a junior high school education level. The questionnaire used in our study has previously been validated and published in previous study (22). The survey instrument of this study consisted of two primary components. The first part gathered demographic information, encompassing gender, age, marital status, occupation, education level, and monthly personal income. To further clarify participants’ experience with cardiopulmonary resuscitation (CPR) as a key aspect of their medical background, this section also included specific items addressing: (1) whether they had received formal CPR training (with options for “never,” “once,” “multiple times”); (2) the time elapsed since their last CPR training (if applicable); and (3) any prior experience of witnessing an out-of-hospital cardiac arrest event or assisting in CPR administration. The second part was the public intention to perform CPR scale (22), which encompassed four dimensions with a total of 42 items. The scale utilized a 5-point Likert scale, where “Strongly Disagree = 1, Disagree = 2, Uncertain = 3, Agree = 4, Strongly Agree = 5”. Specifically, the Behavioral Attitude dimension, with 8 items and a maximum score of 40, indicated a more positive attitude towards performing CPR with higher scores. The Subjective Norm dimension, with 10 items and a maximum score of 50, reflected the extent of perceived social expectation to perform CPR, with higher scores indicating a greater sense of social pressure. The Perceived Behavioral Control dimension, with 15 items and a maximum score of 75, measured the ease with which the public felt they could perform CPR in an out-of-hospital cardiac arrest situation, with higher scores suggesting greater perceived control. The Behavioral Intention dimension, with 9 items and a maximum score of 45, gauged the willingness to perform CPR in an out-of-hospital cardiac arrest scenario, with higher scores indicating a stronger intention to provide aid. The overall scale and each of its dimensions demonstrated high internal consistency, with Cronbach’s *α* coefficients greater than 0.90 and split-half reliability coefficients greater than 0.80, indicating a robust and reliable measurement tool.

### Data collection procedures

Data collection for this study was conducted using the “Wenjuanxing” online survey platform, a widely utilized tool in academic research due to its secure data management features and user-friendly interface. The survey instrument—encompassing the introductory statement (detailing the study purpose, voluntary participation, and data confidentiality protocols) and all questionnaire items—was first uploaded to the Wenjuanxing platform, which generated a unique QR code linked to the survey. Fieldwork was carried out over an 8-week period (May 1 to June 20, 2023) by research team members trained in standardized recruitment protocols. Team members were present at the selected sites during both weekdays and weekends, from 9:00 a.m. to 6:00 p.m., to accommodate varying schedules. Eligible individuals (aged ≥18 years and capable of independent reading) were approached in person; following a brief verbal explanation of the study, interested participants were invited to scan the QR code using their personal devices to access the online questionnaire. A printed information sheet, mirroring the content of the verbal explanation, was provided to all participants for reference.

Upon completion of data collection, raw data were exported from the Wenjuanxing platform in encrypted format. A dual verification process was then implemented: two members of the research team independently reviewed each dataset to cross-check for missing values, logical inconsistencies (e.g., contradictory responses to related items), and abnormal response patterns. Questionnaires were deemed invalid if they met any of the following criteria: a completion time of <1 min (indicating potential inattentiveness), >20% missing data, or evidence of response bias (e.g., identical answers to all items). Invalid records were systematically excluded from subsequent analyses to ensure data quality.

### Statistical analysis

All data were double-entered using EpiData 3.1 and analyzed using SPSS 24.0. Descriptive statistics, including frequencies, means, and standard deviations (SD), were calculated for all study variables. Pearson’s correlation coefficients were used to examine bivariate relationships among the TPB constructs. Multiple stepwise regression analysis was performed to identify predictors of CPR intention. Variance inflation factor (VIF) values were examined to assess multicollinearity, with values below 10 indicating no severe multicollinearity concerns. Structural equation modeling (SEM) was conducted using AMOS 23.0 with maximum likelihood estimation. The initial conceptual model included all theoretically proposed paths among attitude, SN, PBC, and intention, consistent with the TPB framework. Model optimization was guided by modification indices, with a threshold of >10 considered for potential model adjustments; any modifications were evaluated for theoretical plausibility before implementation. Model fit was assessed using multiple indices. Acceptable fit was defined as: ratio of chi-square to degrees of freedom (*χ*^2^/df) < 3.0, root mean square error of approximation (RMSEA) < 0.07, and fit indices including the Goodness of Fit Index (GFI), Incremental Fit Index (IFI), Adjusted Goodness of Fit Index (AGFI), and Comparative Fit Index (CFI) all > 0.90 (23–25). For the final model, exact fit indices were calculated and reported. All statistical tests were two-tailed, with statistical significance set at *p* < 0.05.

## Results

### Participant characteristics

A total of 716 individuals were invited to participate, of whom 682 were enrolled (effective response rate: 95.25%). As shown in Table 1, the sample had a mean age of 38.03 years (SD = 7.11). Among participants, 366 (53.7%) were female, 307 (45.0%) were married, and 300 (44.0%) were self-employed. Significant differences in attitude scores were observed by gender and educational level (both *p* < 0.05), and intention scores differed significantly by educational level (*p* = 0.004).

**Table 1:** Demographic characteristics of participants and comparisons of theory of planned behavior construct scores across subgroups (*N* = 682).

Characteristic	*n* (%)	Attitude (0–40)	*p*-value	Subjective norms (0–50)	*p*-value	Perceived behavioral control (0–75)	*p*-value	Intention (0–45)	*p*-value
		Mean % ± SD		Mean % ± SD		Mean % ± SD		Mean % ± SD	
Gender			**0.01**		0.386		0.983		0.08
Male	316 (46.3)	86.6 ± 21.9		84.1 ± 21.3		83.0 ± 19.5		82.5 ± 23.2	
Female	366 (53.7)	90.3 ± 15.8		85.4 ± 16.8		82.9 ± 15.4		85.2 ± 17.8	
Age (years)			0.278		0.389		0.749		0.928
18–30	300 (44.0)	89.8 ± 17.2		84.5 ± 17.8		82.3 ± 16.4		84.6 ± 19.5	
31–40	130 (19.1)	85.7 ± 20.7		82.6 ± 20.9		82.1 ± 19.4		82.8 ± 21.4	
41–50	167 (24.5)	88.9 ± 19.5		85.5 ± 19.9		84.3 ± 17.5		83.8 ± 21.0	
51–60	73 (10.7)	87.4 ± 21.7		87.2 ± 19.4		83.6 ± 18.4		84.0 ± 22.3	
>60	12 (1.8)	92.5 ± 10.9		90.3 ± 11.7		84.8 ± 12.3		82.0 ± 22.9	
Marital status			0.503		0.360		0.525		0.937
Unmarried	15 (2.2)	89.4 ± 17.6		84.4 ± 18.2		82.7 ± 16.6		84.3 ± 19.9	
Married	660 (96.8)	88.0 ± 19.8		85.4 ± 19.5		83.3 ± 17.7		83.7 ± 21.0	
Divorced	7 (1.0)	85.0 ± 23.4		78.7 ± 24.4		78.4 ± 24.1		84.4 ± 21.8	
Occupation			0.107		0.190		0.366		0.072
Student	21 (3.1)	90.3 ± 16.5		85.2 ± 16.5		82.4 ± 18.1		85.2 ± 19.0	
Company employee	53 (7.8)	88.0 ± 19.8		84.8 ± 19.9		84.0 ± 15.1		83.8 ± 21.2	
Government employee	30 (4.4)	84.2 ± 24.2		79.1 ± 23.4		81.4 ± 19.1		76.6 ± 25.2	
Private owner	300 (44.0)	92.2 ± 19.1		88.4 ± 22.0		79.9 ± 20.1		88.4 ± 22.3	
Freelancer	213 (31.2)	85.7 ± 20.0		84.7 ± 18.9		86.9 ± 20.5		83.2 ± 19.2	
Other	65 (9.5)	93.5 ± 10.2		89.4 ± 15.5		86.4 ± 13.1		88.7 ± 13.6	
Medical background			0.802		0.760		0.915		0.630
No	419 (61.4)	87.7 ± 19.7		84.5 ± 19.8		82.7 ± 21.3		83.3 ± 21.0	
Yes	50 (7.3)	88.5 ± 21.8		85.4 ± 22.3		82.4 ± 18.0		84.8 ± 22.5	
Educational level			**0.032**		0.052		0.098		**0.004**
Junior high school	56 (8.2)	86.6 ± 19.0		86.1 ± 19.6		83.5 ± 18.4		83.4 ± 19.1	
Senior high school	156 (22.9)	92.4 ± 11.6		88.4 ± 15.9		86.5 ± 13.0		88.4 ± 14.3	
Junior college	112 (16.4)	89.2 ± 16.8		85.8 ± 17.6		81.5 ± 17.2		86.0 ± 18.2	
Undergraduate	101 (14.8)	84.5 ± 24.8		81.1 ± 23.4		80.2 ± 21.6		78.7 ± 26.0	
Graduate	44 (6.5)	86.8 ± 22.0		83.3 ± 21.3		82.4 ± 18.3		82.5 ± 22.0	
Monthly income (RMB)			0.092		0.147		0.552		0.062
<5,000	213 (31.2)	86.7 ± 19.5		84.3 ± 20.0		80.7 ± 17.9		83.4 ± 19.7	
5,000–10,000	84 (12.3)	90.0 ± 17.1		86.1 ± 18.5		83.7 ± 18.3		85.6 ± 19.5	
10,000–20,000	51 (7.5)	83.7 ± 25.1		80.3 ± 24.5		82.2 ± 19.7		78.2 ± 25.9	
>20,000	212 (31.1)	88.0 ± 21.2		86.0 ± 18.3		81.8 ± 17.2		83.3 ± 21.7	

Data are presented as mean percentage of maximum possible score ± standard deviation. *p*-values were derived from one-way ANOVA or independent *t*-tests comparing raw mean scores across subgroups. Bolded *p*-values indicate statistical significance (*p* < 0.05). Percentages may not sum to 100 due to missing data for medical background (*n* = 213, 31.3% did not respond). RMB, renminbi (Chinese Yuan); SD, standard deviation.

### Score distributions

Table 2 presents the mean scores for each TPB construct expressed as percentages of the maximum possible score. Participants demonstrated favorable attitudes toward CPR (88.6% ± 18.9%), moderate-to-strong subjective norms (84.8% ± 19.0%), and relatively high perceived behavioral control (82.9% ± 17.4%). The mean intention score was 84.0% ± 20.5% of the maximum possible.

**Table 2:** Mean scores of theory of planned behavior constructs and subdimensions expressed as percentages of maximum possible scores (*N* = 682).

Construct and subdimension	Number of items	Maximum score	Mean % ± SD
Attitude toward CPR	8	40	88.6 ± 18.9
Behavioral beliefs	5	25	88.2 ± 19.0
Evaluations of behavioral outcomes	3	15	89.2 ± 19.5
Subjective norms	10	50	84.8 ± 19.0
Normative beliefs	5	25	84.6 ± 19.4
Compliance motivations	5	25	85.0 ± 19.9
Perceived behavioral control	15	75	82.9 ± 17.4
Self-efficacy	7	35	80.2 ± 19.0
Perceived control	8	40	85.4 ± 17.6
Providing CPR intention	9	45	84.0 ± 20.5
Intention for recipient	3	15	84.5 ± 21.8
Intention for action	6	30	83.7 ± 20.7

Values represent mean percentage of the maximum possible score ± standard deviation. All subdimensions were measured using 5-point Likert scales (1 = strongly disagree to 5 = strongly agree). Higher percentages indicate more favorable attitudes, stronger subjective norms, greater perceived behavioral control, and stronger intentions to perform CPR. CPR, cardiopulmonary resuscitation; SD, standard deviation.

### Correlations analysis on the public intention to perform CPR

**Figure 1:**
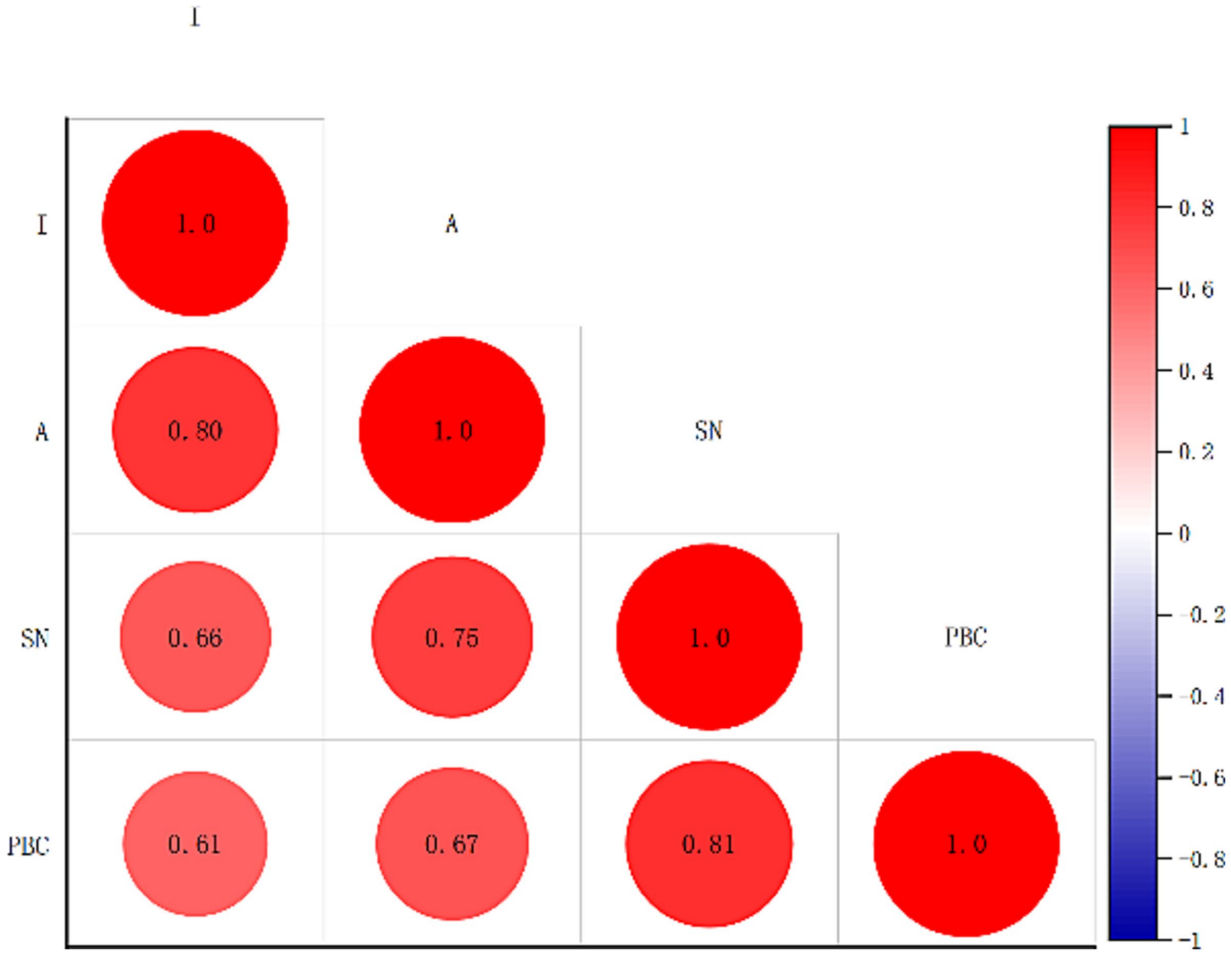
Correlation analysis of behavioral attitude, subjective norms, perceived behavioral control, and behavioral intention toward cardiopulmonary resuscitation (CPR) among 682 participants in a cross-sectional survey conducted in Shanghai, China (May 1–June 20, 2023).

The correlations among the variables within the SEM are depicted in Figure 1. The findings indicated that attitude, SN, and perceived behavioral control PBC were all significantly and positively associated with the public intention to perform CPR (all *r* > 0.60, *p* < 0.05).

As shown in Table 3, multiple stepwise regression analyses had identified attitude and SN as the significant predictors of the public’s intention to perform CPR (all *p* < 0.05).

**Table 3:** Multiple stepwise regression analysis identifying factors influencing public intention to perform CPR, based on data from 682 participants in a cross-sectional survey conducted in Shanghai, China (May 1–June 20, 2023).

DV	IV (variables entered)	Unstandardized coefficients		Standardized coefficients	*t*	Sig.	Collinearity statistics		*F*	*F* change	*R*	*R* square	Adjusted *R* square	*R* square change	Durbin–Watson
		*B*	Standard error	*β*			Tolerance	VIF							
I		0.978	1.113		0.879	0.380									1.987
	A	0.818	0.047	0.683	17.341	<0.001	0.399	2.504	1069.99^***^	1069.99^***^	0.834	0.696	0.696	0.696	
	SN	0.186	0.037	0.196	4.968	<0.001	0.399	2.504	574.457^***^	24.676^***^	0.843	0.711	0.710	0.015	

The table presents unstandardized and standardized coefficients, significance levels, collinearity statistics, and model fit indices (including *R*^2^ and *F* values) for the regression model, which examines the predictive roles of attitude and subjective norms in explaining variance in CPR performance intention.

CPR, cardiopulmonary resuscitation; A, attitude; SN, subjective norm; I, intention; ***p<0.001; **p<0.01; *p<0.05.

### Structural equation modeling

**Figure 2:**
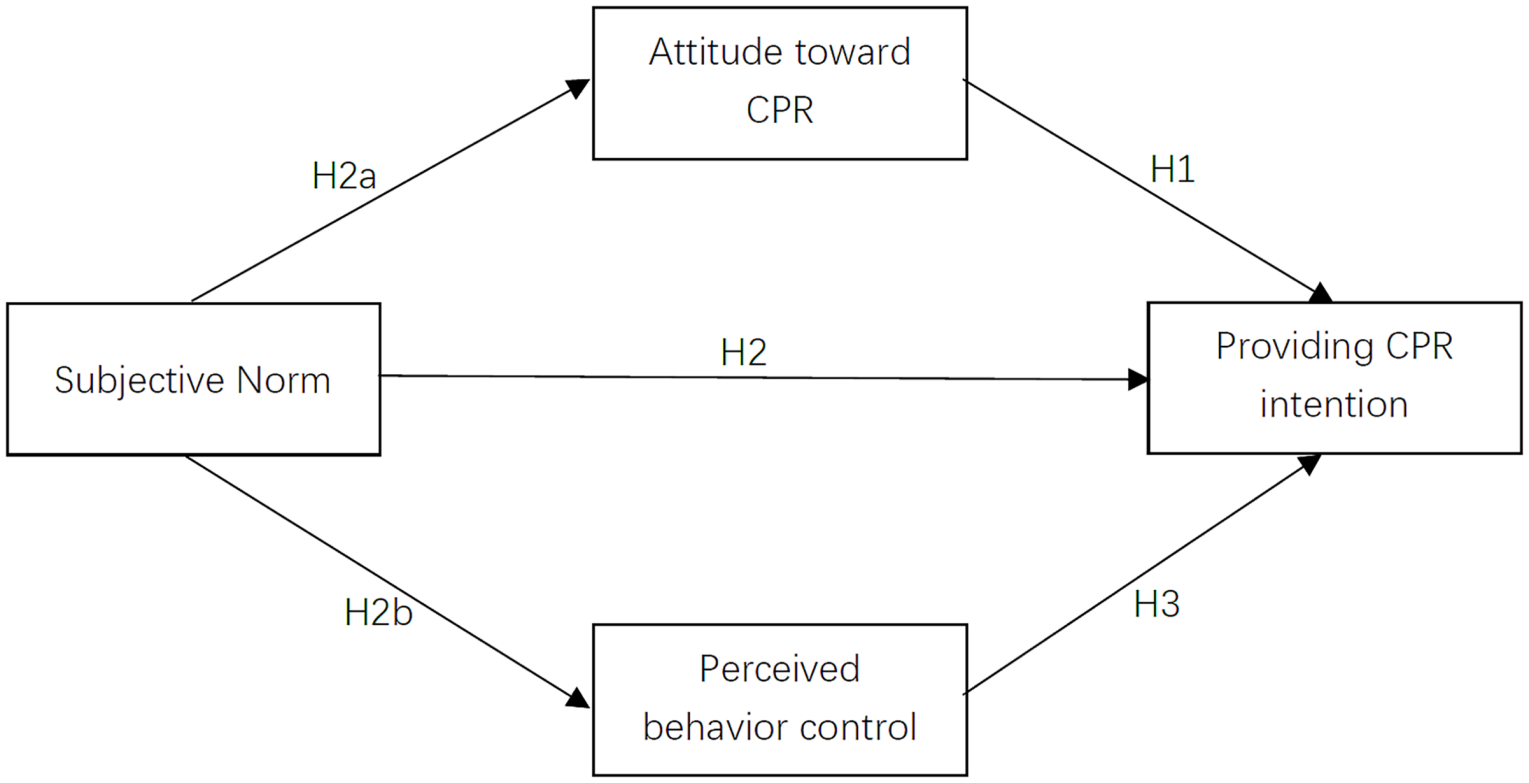
Structural equation model examining relationships between subjective norms, attitude, perceived behavioral control, and CPR performance intention among the general public in Shanghai, China (May 1–June 20, 2023), with standardized path coefficients and model fit indices.

An initial fully saturated model including all theoretically plausible paths was tested (Figure 2). Through iterative optimization guided by modification indices and statistical significance—while remaining grounded in the TPB framework—the final model was refined. The path from attitude to PBC was removed due to non-significance (*β* = 0.05, *p* = 0.18). All retained paths (SN → attitude, SN → PBC, and attitude → intention) are core components of the TPB and remained statistically significant, ensuring that model refinement was not purely data-driven but theoretically justified.

Supplementary Table S1 presents the decomposition of direct, indirect, and total effects among the TPB constructs. While SN demonstrated a negligible direct effect on CPR intention (*β* = 0.027, *p* = 0.715), their indirect effect via attitude and PBC was substantial (*β* = 0.681), yielding a total effect of *β* = 0.708 (95% CI: 0.645–0.765, *p* < 0.001). Attitude remained a strong direct predictor of intention (*β* = 0.732, *p* < 0.001), whereas the direct effect of PBC on intention did not reach statistical significance (*β* = 0.108, *p* = 0.077).

**Figure 3:**
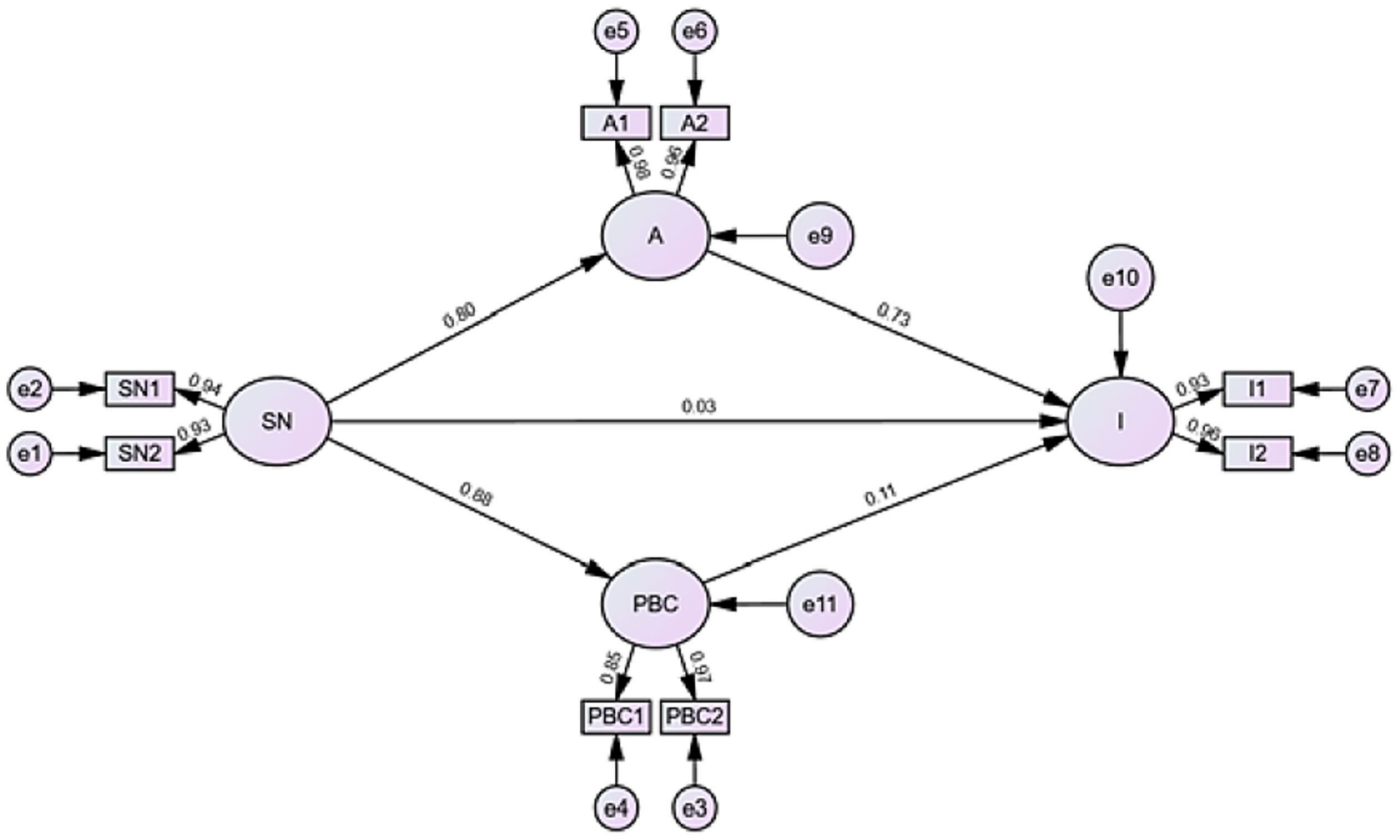
Structural equation model for the public intention to perform CPR.

As shown in Table 4 and Figure 3, attitude emerged as a strong direct predictor of CPR intention (*β* = 0.73, *p* < 0.001). SN exerted substantial positive effects on both attitude (*β* = 0.80, *p* < 0.001) and PBC (*β* = 0.88, *p* < 0.001), indicating that stronger social expectations shape both personal evaluations of CPR and perceived capability to perform it.

**Table 4:** Results of relationship tests using structural equation modeling to examine associations between subjective norms, attitude, perceived behavioral control, and CPR performance intention, based on data from 682 participants in a cross-sectional survey in Shanghai, China (May 1–June 20, 2023).

Approach relationship			Estimate	S.E.	C.R.	*p*
A	←	SN	0.80	0.029	28.170	<0.001
PBC	←	SN	0.88	0.038	33.714	<0.001
I	←	SN	0.03	0.049	0.365	0.715
I	←	A	0.73	0.030	16.107	<0.001
I	←	PBC	0.11	0.027	1.771	0.077

The table includes estimated path coefficients, standard errors, critical ratios, and significance levels for direct relationships between constructs, as derived from the final optimized model.

A, attitude; SN, subjective norm; I, intention; PBC, perceived behavior control; SD, standard deviation.

The final model demonstrated good fit to the data: *χ*^2^/df = 2.87, RMSEA = 0.052, CFI = 0.94, GFI = 0.91, IFI = 0.94, and AGFI = 0.90, all meeting recommended thresholds.

## Discussion

Bystander CPR is critical for improving survival rates and functional outcomes in out-of-hospital cardiac arrest cases, underscoring the urgency of enhancing public readiness to intervene. While global efforts to expand public CPR training have intensified in recent years, a notable gap persists between training uptake and real-world action: prior studies (26, 27) indicate that only 35% of trained individuals actually perform CPR during out-of-hospital cardiac arrest events. This discrepancy highlights that technical training alone may be insufficient to transform knowledge into life-saving behavior, necessitating a deeper understanding of the psychological drivers of bystander action. The TPB provides a framework to address this gap, positing that behavioral intention is the most proximal determinant of actual behavior. Consistent with this, scholars (28, 29) have advocated for reframing public CPR education around the cultivation of rescue intention—an approach that not only promotes immediate intervention but also motivates proactive engagement in CPR learning and knowledge dissemination, thereby accelerating the democratization of life-saving skills. Findings from the present study align with this paradigm, revealing that SN and behavioral attitudes play key roles in shaping the public’s intention to perform CPR. These results suggest that interventions targeting SN—such as amplifying social expectations around bystander responsibility—along with efforts to foster positive attitudes toward CPR (e.g., emphasizing its accessibility and impact), may represent promising strategies to enhance public rescue intention.

Notably, SN exhibited strong associations with attitude and perceived behavioral control (PBC) but only a weak direct link to intention. This pattern reflects nuanced psychological mechanisms in bystander CPR decision-making. Theoretically, it aligns with the TPB framework, in which SN, as a social influence variable, primarily functions by shaping foundational cognitions. Their robust effects on attitude and PBC suggest that social norms reinforce approval of CPR and reduce perceived barriers to action, thereby cultivating pre-behavioral readiness. In contrast, the weak direct association between SN and intention underscores the primacy of personal agency in emergency contexts. Under time pressure, rapid decision-making prioritizes proximal drivers—one’s own evaluative judgments (attitude) and perceived competence (PBC)—over abstract social expectations. Contextually, this aligns with the realities of urban emergency response. In settings such as Shanghai, public health campaigns emphasizing collective responsibility may effectively strengthen SN, fostering favorable attitudes and enhanced PBC. However, the actual decision to intervene hinges on immediate self-assessments of capability and alignment with personal values. Together, these dynamics redefine SN as indirect enablers of intention, operating through attitude and PBC to create the psychological conditions for action. This distinction informs targeted interventions that leverage social norms to reinforce these mediating constructs, bridging the gap between social influence and actionable intent in bystander CPR.

Comprehending and dissecting the patterns and determinants of public involvement in on-site first aid is essential for augmenting the populace’s engagement in immediate rescue operations. Such an endeavor holds profound significance in the construction of an inclusive community-based pre-hospital emergency response framework (30, 31). The findings of this survey indicate that the public holds a correct and positive attitude towards performing CPR, which is influenced by gender and educational level. Females exhibit relatively higher levels of empathy compared to males and are more willing to help others (32). Previous studies (33, 34) have reported that subjects who have participated in CPR theoretical learning and training demonstrate a more pronounced willingness to perform CPR, and these individuals tend to have a higher level of education. Studies (35, 36) have also revealed that the higher the educational level of the public, the stronger their sense of self-efficacy, which helps to reduce perceived barriers; individuals with higher education levels also have a stronger sense of responsibility for advancing national and social progress, and when faced with OHCA events, they may have a more pronounced intention to provide assistance. Therefore, enhancing CPR training, including the popularization of training for the untrained public and regular refresher courses for those who have been trained, is an important means of improving the public’s level of CPR knowledge. In addition, it is necessary to leverage the latest technological means to continuously innovate training models, such as using social media and live broadcasting platforms to expand the reach of CPR training, and developing virtual reality games to teach CPR knowledge and skills in an entertaining way, with the aim of improving the efficiency and effectiveness of CPR training (37–39).

The present study has revealed a significant positive correlation between the public’s SN and their intention to perform CPR, which is consistent with the findings of previous related research (40). Therefore, further exploration of the underlying beliefs that may influence behavioral attitudes and subjective norms is of significant reference and guidance value for optimizing CPR public education and dissemination efforts (41, 42). Previous studies (43, 44) have identified certain beliefs that may adversely affect the public’s intention to perform CPR, such as “fear of causing injury to the patient,” “concern about making mistakes and legal disputes”. Given that mere positive education may not dispel public doubts, CPR public education should focus on targeted explanations of negative beliefs, correcting the public’s misconceptions, and further guiding them positively on this basis, which may truly enhance the public’s sense of identification and responsibility for performing CPR (45). Moreover, it is essential to focus on fostering a culture of mutual aid in emergency situations across society, which will contribute to shaping a positive attitude and social recognition of the significance and value of first aid (46).

*Post-hoc* analyses examining the potential influence of medical background revealed nuanced findings. Although medical background showed a modest, non-significant correlation with perceived behavioral control, it did not significantly moderate the core pathways of the TPB model. Specifically, the relationships between subjective norms and attitude, attitude and intention, and subjective norms and perceived behavioral control remained consistent across participants with and without medical training. This pattern suggests that the psychological mechanisms underlying CPR intention—particularly the central role of subjective norms in shaping attitudes and perceived capability—operate similarly across diverse populations, reflecting overarching cognitive processes captured by the TPB framework that transcend specific training backgrounds. While the small proportion of medically trained individuals (6.3% of the sample) limits statistical power to detect subtle differences, these findings reinforce the generalizability of our model and suggest that interventions targeting subjective norms and attitudes may be broadly effective, with medical background exerting a secondary influence on practical competence rather than altering core psychological drivers of intention.

A notable gap in public health practice emerges from prior research indicating that while most individuals express strong willingness to undergo CPR training, fewer than 50% actually participate in such programs (47, 48). This discrepancy underscores the need for curriculum developers to move beyond generic training designs and instead anchor program refinement in an empirical understanding of public barriers and needs—whether logistical (e.g., time constraints), attitudinal (e.g., perceived irrelevance), or structural (e.g., access to training sites) (49). Tailored training plans that address these specific hurdles are essential to increasing participation rates and expanding the reach of CPR education, thereby strengthening community readiness to respond to cardiac emergencies.

First aid training functions as more than a skill-building tool; it serves as a critical mechanism for dismantling psychological barriers that hinder bystander intervention. By equipping individuals with practical knowledge, training alleviates uncertainty during emergencies, enhances self-efficacy in performing CPR, and reinforces social responsibility—factors that collectively elevate the likelihood of timely first response. Yet, despite high reported willingness to assist during cardiac or respiratory arrest, overall CPR proficiency remains low, creating a paradox between intent and capability. Resolving this paradox requires integrating insights into the root causes of public hesitation—such as fear of causing harm, legal concerns, or lack of confidence—into educational frameworks. Concurrently, leveraging digital platforms to deliver multi-channel, accessible CPR education can overcome traditional barriers to participation, ensuring that knowledge dissemination aligns with contemporary patterns of information consumption. Together, these strategies—contextualized training design and innovative educational outreach—offer a pathway to transforming public willingness into actionable competence, bridging the gap between intent and life-saving action (50–52).

Several limitations should be acknowledged when interpreting the findings of this study. First, the convenience sample recruited from public sites in Shanghai—while strategically selected due to high foot traffic and relevance to out-of-hospital cardiac arrest locations—may limit generalizability. Compared to Shanghai’s general population, our sample over-represented working-age and employed individuals and under-represented older adults and home-bound populations, who may hold different attitudes toward CPR and face distinct barriers to intervention. Findings are therefore most applicable to urban public populations and should be extrapolated cautiously to other settings. Future research should employ multi-center, stratified sampling to enhance external validity. Second, the cross-sectional design captures associations at a single time point, precluding causal inferences about relationships among TPB constructs and CPR intention. Longitudinal or interventional studies are needed to establish temporal precedence and inform causal pathways for targeted interventions. Third, all constructs were assessed using self-report measures, introducing potential social desirability bias. This is particularly relevant for CPR intention, as self-reported willingness often overestimates actual bystander behavior—a well-documented phenomenon in this field. While we mitigated this risk through anonymization and emphasizing voluntary participation, such measures cannot entirely eliminate response inflation. Additionally, prior CPR training and medical background were self-reported and not independently verified. Fourth, several potentially important variables were not included in our model, such as objective CPR knowledge, prior rescue experience, and legal awareness regarding Good Samaritan protections. Their absence may limit the model’s explanatory breadth. Future research should explore extended TPB models incorporating these factors to better capture the complexity of bystander decision-making. Fifth, the 1-min completion threshold used to exclude invalid responses may not have fully ensured thoughtful engagement with the 42-item questionnaire, potentially permitting some inattentive responses. Future studies should adopt multi-center sampling, employ prospective designs to strengthen causal inference, incorporate objective measures to complement self-reported data, and test extended theoretical models that capture additional determinants of bystander CPR intention.

An additional consideration for future research is the potential moderating role of health literacy. Individuals with higher health literacy may be better equipped to translate perceived behavioral control into actual intention, as they possess greater capacity to access, understand, and utilize health information relevant to emergency response. Conversely, those with lower health literacy might experience greater difficulty converting confidence into action, despite holding positive attitudes or perceiving social expectations. While our study did not assess health literacy, its potential to moderate relationships among TPB constructs—particularly the pathway from PBC to intention—warrants investigation. Future studies could extend the TPB framework by incorporating health literacy as a moderating variable, which would enable more nuanced identification of population subgroups requiring tailored intervention approaches. Such insights could inform the design of health literacy-sensitive CPR training programs, ultimately enhancing the effectiveness of community-based emergency preparedness initiatives.

## Conclusion

In summary, the research findings demonstrate that attitude, SN, and PBC are each significantly and positively correlated with the public’s intention to administer CPR. A more favorable attitude is linked to an increased intention to perform CPR, and a robust SN is significantly associated with both a more favorable attitude and an enhanced PBC. These relationships underscore the multifaceted influence of psychological factors on the public’s willingness to engage in life-saving interventions. The TPB offers valuable insights for a deeper comprehension of the public’s intention to perform CPR. Efforts to enhance this intention should adopt a systemic perspective, focusing on the key predictors of CPR practice as identified by TPB. Future initiatives should develop interventions that are specifically tailored to address these predictors, leveraging the theoretical framework of TPB to foster a more proactive and capable public in emergency response scenarios.

## Data Availability

The original contributions presented in the study are included in the article/Supplementary material, further inquiries can be directed to the corresponding author.
