# Supplementary material for: General population’s intentions to perform public CPR: a structural equation modeling analysis based on the theory of planned behavior
Source: Front Public Health. 2026 Apr 13;14:1707826. doi: 10.3389/fpubh.2026.1707826 (PMC13111574; doi:10.3389/fpubh.2026.1707826)
Supplement: Supplementary file 1 [file Supplementary_File_1.docx]

Supplementary file 1 Goodness-of-Fit Indices for the Structural Equation Mode

| Fit Index | Abbreviation | Value | Recommended Threshold¹ | Interpretation |
| --- | --- | --- | --- | --- |
| Chi-square/degrees of freedom | χ²/df | 2.73 | < 3.0 | Indicates acceptable model parsimony |
| Root Mean Square Error of Approximation | RMSEA | 0.058 | < 0.07 | Reflects close fit to the data |
| Goodness-of-Fit Index | GFI | 0.92 | > 0.90 | Measures overall model fit to observed data |
| Adjusted Goodness-of-Fit Index | AGFI | 0.90 | > 0.85 | Adjusts GFI for model complexity, indicating robust fit |
| Comparative Fit Index | CFI | 0.94 | > 0.90 | Compares model fit to a null model, with higher values indicating better fit |
| Incremental Fit Index | IFI | 0.94 | > 0.90 | Assesses incremental improvement over the null model |

¹Thresholds adapted from Hu & Bentler (1999) and Kline (2016) for observational study models.

This table consolidates key indices to demonstrate that the final structural equation model meets or exceeds recommended standards for good fit, supporting the validity of the observed relationships among TPB constructs and CPR intention.
