# Supplementary material for: General population’s intentions to perform public CPR: a structural equation modeling analysis based on the theory of planned behavior
Source: Front Public Health. 2026 Apr 13;14:1707826. doi: 10.3389/fpubh.2026.1707826 (PMC13111574; doi:10.3389/fpubh.2026.1707826)
Supplement: Supplementary file 2 [file Table_1.docx]

Supplementary Table 1 Decomposition of direct, indirect, and total effects for the final structural equation model

| Predictor | Outcome | Direct effect β | Indirect effect β | Total effect β | 95% CI for total effect | P-value |
| --- | --- | --- | --- | --- | --- | --- |
| SN | Attitude | 0.800 | — | 0.800 | (0.743, 0.852) | <0.001 |
| SN | PBC | 0.880 | — | 0.880 | (0.831, 0.924) | <0.001 |
| SN | Intention | 0.027 | 0.681 | 0.708 | (0.645, 0.765) | <0.001 |
| Attitude | Intention | 0.732 | — | 0.732 | (0.678, 0.781) | <0.001 |
| PBC | Intention | 0.108 | — | 0.108 | (0.031, 0.184) | 0.077 |

*Note*: Standardized path coefficients (β) are presented. Indirect effects were calculated as the product of constituent paths. For SN → Intention, the indirect effect comprises two pathways: via attitude (SN → Attitude × Attitude → Intention = 0.800 × 0.732 = 0.586) and via PBC (SN → PBC × PBC → Intention = 0.880 × 0.108 = 0.095), summing to 0.681. Confidence intervals were estimated using bootstrapping with 5,000 resamples. Abbreviations: SN, subjective norms; PBC, perceived behavioral control; CI, confidence interval.
